# Sectoral Analysis of Corneal Thickness in Glaucoma and Healthy Eyes and Its Relationship with RNFL and Rim Area

**DOI:** 10.3390/jcm15062405

**Published:** 2026-03-21

**Authors:** Piotr Miklaszewski, Anna Maria Gadamer, Zuzanna Lelek, Dominika Janiszewska-Bil, Anita Lyssek-Boroń, Dariusz Dobrowolski, Edward Wylęgała, Beniamin Oskar Grabarek, Michael Janusz Koss, Katarzyna Krysik

**Affiliations:** 1 Department of Ophthalmology, St. Barbara Hospital, Trauma Centre, 41-200 Sosnowiec, Poland; ania.gadamer@gmail.com (A.M.G.); zuzkalelek@gmail.com (Z.L.); dominika.bjaniszewska@gmail.com (D.J.-B.); anitaboron3@gmail.com (A.L.-B.); dardobmd@wp.pl (D.D.); kkrysik@gmail.com (K.K.); 2 Department of Ophthalmology, Faculty of Medicine, Academy of Silesia, 40-555 Katowice, Poland; 3 Collegium Medicum, WSB University, 41-300 Dabrowa Gornicza, Poland; bgrabarek7@gmail.com; 4 Department of Ophthalmology, District Railway Hospital, 40-760 Katowice, Poland; wylegala@sum.edu.pl; 5 Department of Ophthalmology, Faculty of Medicine, Medical University of Silesia, 40-555 Katowice, Poland; 6 Augenzentrum Nymphenburger Höfe, 80335 Munich, Germany; michael.koss@me.com; 7 Department of Ophthalmology, Augenklinik der Universität Heidelberg, 69120 Heidelberg, Germany; 8 Augenklinik Herzog Carl Theodor, 80335 Munich, Germany

**Keywords:** primary open-angle glaucoma, corneal thickness, retinal nerve fiber layer, optic disc, optical coherence tomography

## Abstract

**Background/Objectives**: To characterize sectoral corneal thickness (CT) profiles in eyes with primary open-angle glaucoma (POAG) compared with healthy eyes and to evaluate potential associations between CT, retinal nerve fiber layer (RNFL) thickness, and optic disc rim area (RA). **Methods**: In this cross-sectional study, 192 participants (91 with POAG and 101 controls) contributed 297 eyes (145 glaucoma eyes and 152 control eyes). All participants underwent comprehensive ophthalmological examination and spectral-domain optical coherence tomography (OCT; Optovue Solix, Fremont, CA, USA) to obtain peripapillary RNFL measurements, optic disc rim area, and corneal pachymetry maps across five sectors (central, superior, inferior, temporal, and nasal). Repeated-measures correlation analyses were used to assess within-subject associations between CT and RA, and generalized estimating equation (GEE) models were applied to evaluate independent associations between CT, glaucoma status, disease severity, and RNFL thickness while adjusting for relevant covariates. **Results**: Eyes with POAG exhibited significantly thinner corneas across all sectors compared with controls (all *p* < 0.05), with the greatest differences observed in the superior (median 607.0 μm vs. 640.0 μm, *p* < 0.001) and temporal (562.0 μm vs. 579.5 μm, *p* < 0.001) regions. Average RNFL thickness and rim area were also significantly reduced in glaucoma eyes (all *p* < 0.001). However, no independent associations between sectoral CT and RNFL thickness or RA were observed after adjustment for multiple comparisons. Although nominal associations between thinner inferotemporal CT and reduced RNFL thickness were observed in unadjusted analyses, these did not remain statistically significant after false discovery rate correction. In multivariable GEE models, glaucoma diagnosis and greater disease severity were consistently associated with reduced RNFL thickness (β range: −11.0 to −42.2 μm; all *p* < 0.001), whereas CT was not independently associated with RNFL thickness (all adjusted *p* > 0.07). **Conclusions**: Sectoral corneal thickness is significantly reduced in eyes with POAG but does not independently correlate with RNFL thickness or optic disc rim area after adjustment for confounding factors. These findings support the concept that corneal thinning reflects structural and biomechanical susceptibility to glaucoma rather than serving as a marker of established neuroretinal damage severity. Further longitudinal studies incorporating comprehensive biomechanical assessments are warranted to clarify the role of corneal structure in glaucoma pathophysiology.

## 1. Introduction

Glaucoma is an optic neuropathy characterized by progressive degeneration of retinal ganglion cells [1]. It is the leading cause of irreversible blindness worldwide [1]. Currently, more than 12 million people in Europe are affected by glaucoma, with nearly half of them remaining undiagnosed [2].

The diagnosis of glaucoma includes measurement of intraocular pressure (IOP), assessment of the optic nerve head, retinal nerve fiber layer (RNFL), the ganglion cells complex (GCC) using OCT, and visual field testing [3,4].

Structural parameters of the optic nerve head, such as RNFL thickness and rim area (RA), are important markers of glaucomatous damage. Their progressive reduction occurs in parallel with disease progression and visual field deterioration [5].

These structural alterations precede the onset of visual field defects, and the rate of their loss closely correlates with the risk of glaucoma progression [6,7].

Moreover, measurements of retinal nerve fiber layer (RNFL) thickness obtained using OCT show excellent repeatability and stability in both healthy individuals and glaucoma patients, even in advanced stages of the disease. The mean RNFL thickness is considered the most reliable parameter for monitoring longitudinal changes over time [5,8].

One of the primary risk factors for glaucoma development is elevated IOP, although the disease can also progress in individuals with normal IOP levels [3,9,10,11].

The accuracy of IOP measurement depends on the biomechanical properties of the cornea, particularly its central thickness (CCT). A thinner cornea underestimates IOP readings, whereas a thicker one overestimates them, which may affect the diagnosis and assessment of glaucoma progression. This effect is especially evident in Goldmann applanation tonometry (GAT), considered the gold standard for IOP measurement [12,13,14,15]. Accurate measurement of CCT is important for evaluating intraocular pressure and planning corneal procedures. It can be assessed using ultrasound pachymetry, tonopachymetry, AS-OCT, or Scheimpflug imaging [16,17].

Ultrasound pachymetry is a traditional method that uses sound waves to measure corneal thickness. It offers high repeatability, although contact between the probe and the cornea may cause measurement errors [18,19]. This method requires the use of local anesthesia and carries the risk of corneal epithelial damage and infection. For these reasons, non-contact techniques such as OCT are gaining popularity, as they provide greater precision while eliminating the risks associated with direct corneal contact [19].

Spectral-domain optical coherence tomography (SD-OCT) is recognized as one of the most precise and repeatable methods for measuring central corneal thickness (CCT) [20]. A strong agreement has been demonstrated between SD-OCT and ultrasound pachymetry, confirming the high accuracy of both methods [21]. Therefore, SD-OCT represents a reliable and noninvasive alternative to conventional ultrasound pachymetry in the assessment of corneal thickness.

Central corneal thickness has been extensively investigated as a risk factor for glaucoma progression and as a structural biomarker associated with optic nerve head morphology and glaucomatous damage. Recent studies confirm correlations between CCT and optic nerve structural parameters, including cup-to-disc ratio and cup volume, as well as biomechanical properties related to glaucoma severity [22,23,24]. Although structural alterations in glaucoma often occur in localized sectors of the optic nerve head and RNFL, most prior investigations have focused on global corneal thickness rather than regional corneal variations, despite the well-recognized regional heterogeneity of glaucomatous structural damage [25,26]. Furthermore, RNFL thickness is influenced by multiple anatomical and demographic variables, including age, axial length, and structural optic nerve characteristics, highlighting the importance of multivariable models when evaluating structural relationships [27,28]. Notably, few contemporary studies have simultaneously evaluated corneal thickness and RNFL thickness using multivariable approaches that account for disease severity and structural optic nerve parameters, underscoring the novelty and clinical relevance of the present analysis.

Therefore, the aim of this study was to comprehensively evaluate sectoral corneal thickness across five anatomical regions using OCT-based pachymetry and to assess its relationship with retinal nerve fiber layer thickness and optic disc rim area in both glaucoma patients and healthy controls. In addition, we examined whether corneal thickness independently predicts structural optic nerve changes after adjustment for relevant clinical and demographic factors. By integrating sectoral pachymetric analysis with multivariable structural modeling, this study seeks to clarify whether corneal thickness reflects biomechanical susceptibility to glaucoma or serves as a marker of established neurodegenerative damage.

## 2. Materials and Methods

### 2.1. Study Design and Ethical Approval

This cross-sectional study included 192 participants (91 with glaucoma and 101 healthy controls), contributing a total of 297 eyes for analysis (145 glaucoma eyes and 152 control eyes). Participants were consecutively recruited from the Ophthalmology Department of Saint Barbara Hospital, Trauma Center in Sosnowiec, between 2023 and 2025.

The study protocol was approved by the Institutional Ethics Committee (No. 24/KB/AŚ/04/2024) and was conducted in accordance with the tenets of the Declaration of Helsinki. Written informed consent was obtained from all participants prior to enrollment.

### 2.2. Study Population and Group Classification

Participants were divided into two groups based on clinical examination and optical coherence tomography (OCT) criteria. The glaucoma group (study group) included eyes with clinically confirmed primary open-angle glaucoma and ongoing antiglaucoma treatment for a minimum of 12 months prior to enrollment. In addition, all glaucoma eyes fulfilled both of the following OCT criteria: global average retinal nerve fiber layer (RNFL) thickness below the 5th percentile, indicated as yellow or red in the Optovue Solix report, and at least one RNFL sector below the 5th percentile.

The control group included eyes with all peripapillary RNFL measurements, including the global average and all four quadrants (superior, temporal, inferior, and nasal), within the normal range (≥5th percentile), with all sectors marked green in the Optovue Solix report. Only eyes without clinical or perimetric signs of glaucoma and with intraocular pressure within the normal physiological range (10–21 mmHg) were included in this group.

All participants underwent a comprehensive ophthalmological evaluation, including best-corrected distance visual acuity (BCVA), intraocular pressure (IOP) measurement using non-contact air-puff tonometry, slit-lamp examination of the anterior segment, and dilated fundus examination. To avoid any potential alteration of corneal surface integrity that could affect pachymetric measurements, contact tonometry methods, including Goldmann applanation tonometry, were not performed.

Eyes were excluded if any condition was present that could affect the accuracy, reliability, or comparability of corneal or RNFL measurements. Specifically, exclusion criteria included a history of ocular surgery, such as refractive procedures, corneal surgery, transplantation, or cataract surgery performed within six months prior to examination. Eyes with active or previous ocular inflammation, trauma, or corneal pathology—including corneal dystrophies, degenerations, keratoconus, scarring, or edema—were also excluded to prevent distortion of pachymetric measurements. Additionally, eyes with clinically diagnosed dry eye disease, meibomian gland dysfunction, or recent soft contact lens wear within one week prior to examination were excluded, as these conditions may transiently alter corneal hydration and thickness. Eyes with non-glaucomatous optic neuropathies, including ischemic, compressive, inflammatory, hereditary, or congenital forms, as well as eyes with tilted optic discs or optic disc drusen, were excluded to ensure accurate structural evaluation of the optic nerve. Finally, eyes with high myopia associated with myopic retinal atrophy or degenerative changes were excluded due to the potential impact on RNFL measurement reliability.

Detailed longitudinal treatment duration and medication class–specific exposure data were not consistently available for all patients; therefore, treatment exposure was indirectly accounted for through inclusion of disease severity and glaucoma diagnosis as covariates in multivariable models.

### 2.3. Optical Coherence Tomography Imaging and Measurements

Optical coherence tomography imaging was performed using the Optovue Solix system (Optovue Inc., Fremont, CA, USA), which operates at a scanning speed of 120,000 A-scans per second and provides high-resolution imaging of both anterior and posterior segment structures.

The device enables detailed visualization and quantitative assessment of corneal pachymetry, corneal epithelial and stromal thickness, anterior chamber structures, retinal layers, RNFL thickness, ganglion cell complex (GCC), and optic nerve head parameters.

All OCT scans were reviewed for segmentation accuracy and image quality. Only scans with a signal strength of at least 7/10 were included in the analysis. Optic nerve head parameters, including rim area (RA), were obtained from the standard Optovue report, which provides automated quantitative measurements based on the manufacturer’s integrated normative database.

### 2.4. Statistical Analysis

Demographic and ophthalmological characteristics were summarized for the overall cohort and stratified by study group (glaucoma vs. control). Continuous variables were described using medians with interquartile ranges (Q1, Q3) and full ranges (minimum–maximum), while categorical variables were presented as counts (n) and percentages (%).

Confidence intervals (95% CI) for continuous variables were calculated using Wilcoxon-based non-parametric methods. This approach was selected due to the non-normal distribution of most continuous variables, as confirmed by the Shapiro–Wilk test (Table S1). Rank-based confidence interval estimation does not assume normality and provides robust inference for skewed biomedical data, including OCT-derived structural measurements.

Group comparisons were performed using the Wilcoxon rank-sum test for continuous variables and Pearson’s chi-squared test for categorical variables, with Fisher’s exact test applied where appropriate.

Normality of ophthalmological parameters was assessed using the Shapiro–Wilk test stratified by group (Table S1), which demonstrated significant deviations from normality (*p* < 0.05) for most variables. Therefore, non-parametric statistical methods were used throughout the analysis.

To evaluate the relationships between rim area (RA) and corneal thickness (CT) parameters (superior, temporal, inferior, nasal, and central CT), repeated-measures correlation analyses were performed separately for glaucoma and control groups [29].

To minimize potential confounding, multivariable generalized estimating equation (GEE) models were constructed, incorporating age, sex, and eye laterality as covariates. Age was included as a continuous variable to preserve biological variability and reduce residual confounding. In addition, glaucoma diagnosis and disease severity were included as independent predictors to account for cumulative disease burden and treatment exposure.

This modeling strategy allowed estimation of independent associations between corneal thickness and RNFL parameters while accounting for intra-subject correlation and relevant clinical covariates.

All statistical analyses were conducted using R Statistical Software (version 4.3.3; R Core Team, 2024, Vienna, Austria) on Windows 11 Pro 64-bit. The following packages were used: parameters (version 0.22.2), report (version 0.5.8), gtsummary (version 2.2.0), glmtoolbox (version 0.1.12), rmcorr (version 0.7.0), and dplyr (version 1.1.4).

Statistical significance was defined as a two-sided *p*-value < 0.05.

## 3. Results

### 3.1. Demographic Characteristics of the Study Population

The study cohort comprised 192 participants (91 with glaucoma and 101 controls), contributing 297 eyes for analysis (145 glaucoma eyes and 152 control eyes). At the patient level, the overall median age was 70 years, with glaucoma patients being significantly older (median 72 years) than controls (median 66 years; *p* < 0.001), highlighting the strong association between increasing age and glaucoma occurrence. Sex distribution was comparable between groups (approximately 56% female overall), with no statistically significant difference (*p* = 0.729). Detailed demographic characteristics of the study population are presented in Table 1.

**Figure 1:**
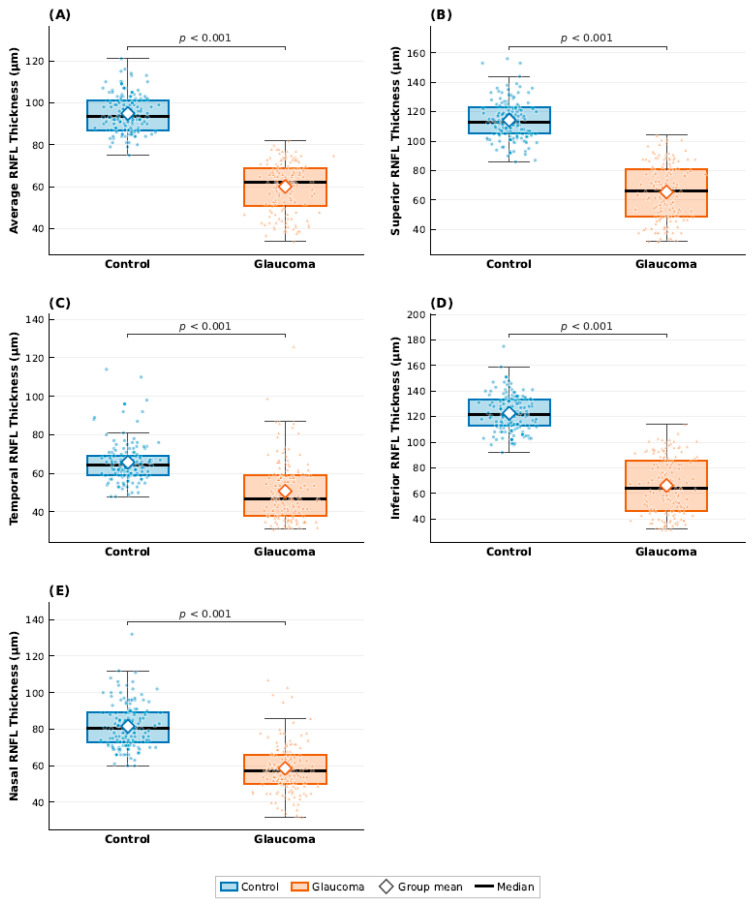
Boxplots comparing retinal nerve fibre layer (RNFL) thickness between glaucoma and control eyes. Panels display (**A**) average, (**B**) superior, (**C**) temporal, (**D**) inferior, and (**E**) nasal RNFL thickness. Boxes represent the interquartile range (IQR) with the median indicated by a horizontal black line; whiskers extend to 1.5 × IQR. Individual observations are overlaid as jittered points, and group means are denoted by open diamond markers. *p*-values were derived from the Wilcoxon rank-sum test.

At the eye level, glaucoma eyes demonstrated marked structural differences compared with control eyes, particularly in optic nerve and RNFL parameters. Glaucoma eyes exhibited significantly reduced rim area and substantially lower RNFL thickness across all measured sectors, including average, superior, temporal, inferior, and nasal regions (all *p* < 0.001). These differences are visually illustrated in Figure 1, which demonstrates a clear shift toward lower RNFL thickness values in glaucoma eyes, with distinct separation of median and distribution ranges between groups.

**Figure 2:**
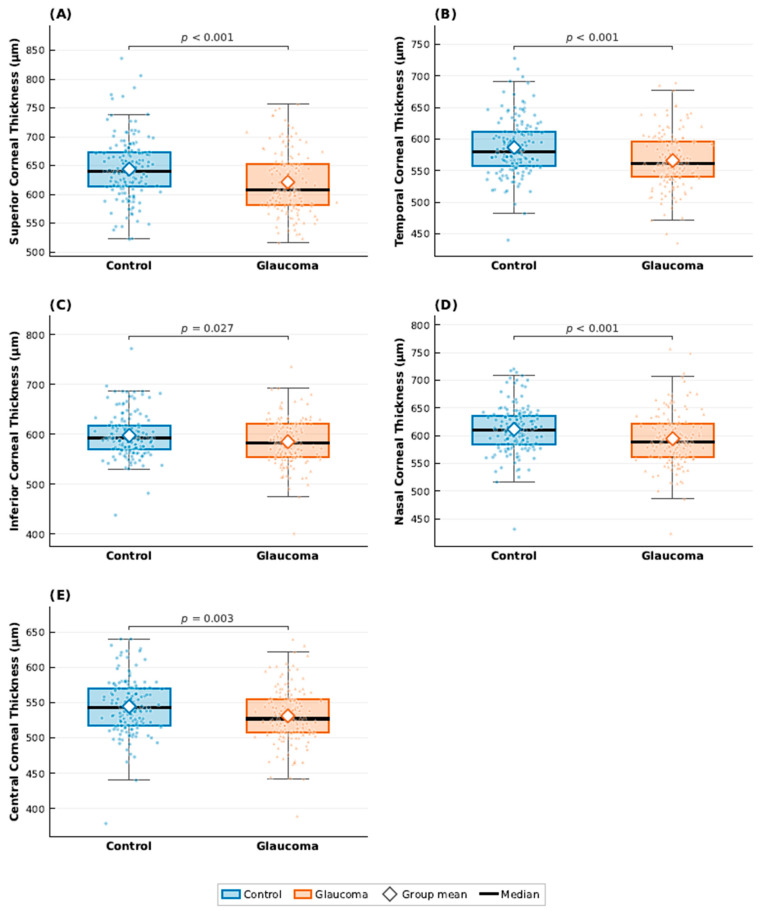
Boxplots comparing sectoral corneal thickness between glaucoma and control eyes. Panels display (**A**) superior, (**B**) temporal, (**C**) inferior, (**D**) nasal, and (**E**) central corneal thickness. Boxes represent the interquartile range (IQR) with the median indicated by a horizontal black line; whiskers extend to 1.5 × IQR. Individual observations are overlaid as jittered points, and group means are denoted by open diamond markers. *p*-values were derived from the Wilcoxon rank-sum test.

Similarly, corneal thickness measurements revealed consistently lower values in glaucoma eyes across multiple sectors. Significant differences were observed in superior, temporal, nasal, and central corneal thickness (all *p* ≤ 0.003), with a smaller but still statistically significant difference in the inferior sector (*p* = 0.027). These sectoral variations are presented graphically in Figure 2, which highlights the systematic reduction in corneal thickness in glaucoma eyes compared with controls, along with group variability and individual measurement distributions.

Detailed numerical ophthalmological parameters at the eye level are presented in Table A1 (Appendix A), while Figure 1 and Figure 2 illustrate the distributional differences between glaucoma and control eyes.

### 3.2. Ophthalmological Characteristics and Group-Level Comparisons

At the eye level, optic disc and retinal parameters revealed marked structural differences consistent with glaucomatous damage. Rim area was substantially reduced in glaucoma eyes (median 0.8 mm^2^) compared to controls (1.5 mm^2^; *p* < 0.001), underscoring optic nerve head atrophy as a hallmark feature that could aid in early detection and staging during clinical examinations. RNFL thicknesses were uniformly thinner in glaucoma across all sectors (e.g., average RNFL: 62 μm vs. 93.5 μm; all *p* < 0.001), reflecting axonal loss that associates with visual field defects.

Corneal thickness (CT) parameters also differed significantly, with glaucoma eyes showing thinner measurements in most regions (e.g., superior CT: 607 μm vs. 640 μm; *p* < 0.001), potentially indicating biomechanical contributions to glaucoma pathogenesis, such as thinner cornea leading to underestimated intraocular pressure readings and increased susceptibility to optic nerve damage. However, the inferior CT difference was less pronounced (*p* = 0.027), which may indicate regional variability in corneal involvement, offering insights for targeted imaging in advanced cases. Within the glaucoma group, 55.9% (n = 81) of eyes exhibited severe disease, aligning with expected distributions in tertiary care settings and highlighting the need for stratified management strategies to prevent irreversible vision loss.

### 3.3. Sectoral Patterns of Retinal and Corneal Thickness

In the control group, the ISNT rule (Inferior > Superior > Nasal > Temporal RNFL thickness) was preserved based on median values (121.5 > 113.0 > 80.5 > 64.5 μm), confirming normal retinal structure. In contrast, the glaucoma group violated the rule (64.0 < 66.0 > 57.0 > 47.0 μm), with superior RNFL exceeding inferior, validating the presence of glaucomatous damage in the study cohort.

### 3.4. Associations Between Rim Area and Corneal Thickness

The repeated measures correlations between rim area and corneal thickness parameters were estimated separately for the glaucoma and control groups, accounting for within-patient clustering. According to findings in Table 2, in the glaucoma group, correlations between RA and CT parameters were generally weak and non-significant, except for a borderline negative association with temporal CT (r = −0.264, 95% CI: −0.495, 0.001; *p* = 0.051), where reduced RA corresponded to thinner temporal CT. Superior, inferior, nasal, and central CT showed negligible correlations (r ranging from −0.139 to 0.034; all *p* > 0.300), indicating limited relationships in this subgroup. In the control group, all correlations were weak and non-significant (r ranging from −0.154 to 0.138; all *p* > 0.270), consistent with expected stability in healthy optic structures. Overall, RA and CT associations aggregated to near-zero across groups, with no consistent patterns of elevation or reduction in correlations, supporting independence of these parameters in both diseased and healthy eyes.

### 3.5. Multivariable Analysis of RNFL Determinants

In the overall sample glaucoma diagnosis was independently associated with thinner RNFL across all sectors and average measurements (adjusted β −11.0 to −42.2 μm; all *p*_adj_ < 0.001), with the inferior sector showing the greatest magnitude of reduction (β −42.2 μm). This consistent finding supports clinical recognition of glaucoma as a primary driver of RNFL attenuation, particularly in vulnerable inferior regions, which may guide prioritization of sectoral imaging in diagnostic protocols to detect early progression.

Rim area was positively associated with RNFL thickness in eight models (adjusted β 4.53 to 13.45 μm; *p*_adj_ < 0.001 to 0.014), indicating that larger rim areas correlate with preserved RNFL integrity and potentially better optic nerve resilience. The non-significant association for nasal RNFL (*p*_adj_ = 0.114) may reflect anatomical differences in fiber density, indicating clinicians consider sectoral variations when evaluating disc-RNFL relationships for risk stratification.

Corneal thickness main effects were non-significant across models (all *p*_adj_ > 0.730), revealing no substantial direct influence on RNFL independent of glaucoma status. Interaction terms (CT × group) were initially suggestive for temporal, inferior, and central CT in unadjusted analyses (*p* = 0.042, 0.016, 0.096) but non-significant after FDR adjustment (*p*_adj_ = 0.126, 0.074, 0.171), implying that corneal thickness-RNFL associations do not differ between glaucoma and control eyes. This lack of interaction reinforces the utility of corneal pachymetry primarily for intraocular pressure correction and biomechanical risk assessment rather than direct RNFL prediction in mixed cohorts.

Age, sex, and eye laterality, which were included as covariates to control for potential demographic confounding, demonstrated no significant independent associations with RNFL thickness after multivariable adjustment (all *p*_adj_ > 0.170), confirming that the observed structural differences were primarily attributable to glaucoma-related factors rather than age-related effects. These findings support the conclusion that sectoral corneal thickness does not independently predict RNFL thickness after adjustment for confounding factors and should be interpreted as exploratory structural observations rather than confirmatory predictors of glaucomatous neurodegeneration.

### 3.6. Effects of Disease Severity on RNFL and Structural Parameters

In the glaucoma subgroup severe disease status was uniformly associated with reduced RNFL thickness across all outcomes (adjusted β range −11.5 to −19.8; all *p*_adj_ < 0.001), with the most substantial reductions observed in superior and inferior sectors (β −18.7 μm and −19.8 μm, respectively). This finding underscores the progressive axonal degeneration in advanced glaucoma, supporting clinical prioritization of severity staging for prognostic assessment.

Rim area showed positive associations with RNFL thickness in models examining average RNFL with superior CT, temporal RNFL with temporal CT, and inferior RNFL with inferior CT (β 6.5 to 8.7; *p* = 0.026 to 0.110, *p*_adj_ = 0.153 to 0.360), though these did not attain significance after adjustment, demonstrating a potential but inconsistent protective role of preserved disc structure in sectoral RNFL integrity that merits further exploration in larger cohorts. Corneal thickness main effects were non-significant (β range −0.03 to 0.06; all *p*_adj_ > 0.150), indicating no substantial direct influence on RNFL within this subgroup. Interaction terms (CT × severe glaucoma) were also non-significant (all *p*_adj_ > 0.150), implying that corneal thickness’s relationship with RNFL does not vary by disease severity.

Age exhibited isolated associations approaching significance in select models (e.g., nasal RNFL with nasal CT; *p* = 0.030, *p*_adj_ = 0.153), but overall non-significant effects for age, sex, and eye laterality (all *p*_adj_> 0.150) affirm their minimal confounding impact in advanced glaucoma.

These results highlight disease severity as the dominant driver of RNFL thinning, with limited contributions from corneal thickness or rim area, informing clinical strategies focused on early intervention to prevent optic nerve damage.

Corneal thickness was significantly lower in glaucoma eyes compared to healthy controls across all examined sectors (superior, temporal, nasal, inferior, and central). The greatest thinning was observed in the superior and temporal regions, whereas the inferior sector showed the smallest difference (all *p* < 0.05)

In the overall sample, glaucoma diagnosis was consistently associated with reduced RNFL thickness across all sectors and average measurements (adjusted β −11.0 to −42.2 μm; all *p*_adj_ < 0.001), with the inferior sector showing the greatest reduction (β −42.2 μm). Rim area was positively associated with RNFL thickness in eight models (adjusted β 4.5 to 13.5 μm; *p*_adj_ < 0.001 to 0.014), except for nasal RNFL (*p*_adj_ = 0.114). Corneal thickness (CT) main effects and CT × group interactions were non-significant (all *p*_adj_ > 0.07), indicating no direct or group-specific influence on RNFL.

In the glaucoma subgroup, severe disease was linked to thinner RNFL across all outcomes (adjusted β −11.5 to −19.8 μm; all *p*_adj_ < 0.001), particularly in superior and inferior sectors. Rim area showed positive but non-significant associations in three models (*p*_adj_ > 0.15), while CT main effects and interactions with severity were non-significant (all *p*_adj_ > 0.15).

Age, sex, and eye laterality had negligible effects (all *p*_adj_ > 0.15).

### 3.7. Summary of Secondary and Supplementary Analyses

Secondary analyses showed weak, non-significant correlations between rim area and CT (r −0.26 to 0.14; all *p* > 0.05), indicating independent structural roles.

Detailed statistical data, including full results of the GEE models and sectoral correlation analyses, are presented in the Supplementary Material (Tables S1–S20).

## 4. Discussion

In this study, we performed a comprehensive sectoral analysis of corneal thickness and its relationship with retinal nerve fiber layer (RNFL) thickness and optic disc rim area in patients with primary open-angle glaucoma and healthy controls. The principal findings can be summarized as follows: (1) corneal thickness was significantly reduced across all sectors in glaucoma eyes compared with controls; (2) glaucoma diagnosis and disease severity were strongly associated with reduced RNFL thickness; and (3) corneal thickness, whether assessed globally or sectorally, did not demonstrate independent associations with RNFL thickness after multivariable adjustment and correction for multiple testing. These findings suggest that while reduced corneal thickness represents a structural characteristic associated with glaucoma, it does not directly reflect the current degree of neuroretinal degeneration. Instead, corneal thickness appears to function primarily as a susceptibility marker rather than a surrogate indicator of ongoing axonal loss. Importantly, the multivariable modeling approach incorporated age, sex, eye laterality, and rim area as covariates, minimizing confounding effects from demographic and anatomical variability. Age was included as a continuous variable to preserve biological variability and reduce residual confounding. Consistent with this adjustment strategy, age was not independently associated with RNFL thickness after correction for multiple testing, indicating that the observed differences were primarily attributable to glaucoma-related structural damage rather than age alone. However, because glaucoma patients were significantly older than controls, residual confounding cannot be entirely excluded. Aging is known to influence both RNFL thickness and corneal structure, with progressive axonal loss and alterations in extracellular matrix composition occurring over time [5]. Therefore, although statistical adjustment mitigates this effect, longitudinal age-matched studies would provide additional confirmation.

The rationale for evaluating sectoral corneal thickness in relation to RNFL parameters is based on the concept that the cornea, sclera, and lamina cribrosa share common extracellular matrix composition and biomechanical properties. These interconnected structures collectively determine the eye’s biomechanical response to intraocular pressure—related stress. Regional variations in corneal thickness may therefore reflect differences in tissue stiffness and structural susceptibility to glaucomatous damage. However, in the present study, sectoral corneal thickness did not demonstrate independent associations with RNFL thickness after multivariable adjustment, indicating that corneal thickness may serve primarily as a marker of biomechanical susceptibility rather than a direct indicator of neuroretinal damage severity.

The observed reduction in corneal thickness in glaucoma patients is consistent with prior evidence demonstrating that thinner corneas are associated with increased risk of glaucoma development and progression [12,13,14,30,31,32]. Several mechanisms may underlie this association. Corneal thickness reflects the structural integrity and biomechanical properties of ocular connective tissues, including the sclera and lamina cribrosa, which share similar collagen and elastin architecture [33]. Reduced corneal thickness may therefore reflect a generalized biomechanical phenotype characterized by increased tissue deformability and vulnerability to chronic intraocular pressure–related stress. This concept is supported by studies demonstrating that lower corneal hysteresis, a measure of corneal viscoelastic damping capacity, is associated with accelerated glaucoma progression and increased susceptibility to optic nerve damage [34,35,36,37].

It is important to emphasize, however, that in our study corneal thickness did not independently predict RNFL thickness after adjustment for glaucoma status and disease severity. Although unadjusted analyses suggested weak inferotemporal associations, these did not remain statistically significant after false discovery rate correction. Therefore, these findings should be interpreted as exploratory and hypothesis-generating rather than confirmatory. The absence of independent associations in multivariable models indicates that corneal thickness does not directly reflect the extent of established neurodegeneration but instead may represent a predisposing structural characteristic.

The sectoral analysis performed in this study was motivated by the hypothesis that regional variations in corneal thickness might correspond to localized differences in biomechanical susceptibility. Because the cornea, sclera, and lamina cribrosa function as a biomechanical continuum, regional variations in tissue thickness may theoretically influence stress transmission to the optic nerve head [33,34,38,39,40,41]. However, the lack of statistically significant sector-specific associations after correction suggests that such regional relationships, if present, are likely subtle and require further investigation using larger cohorts and direct biomechanical measurements.

Alternative analytical frameworks may provide additional insight. Global corneal thickness indices, composite biomechanical parameters, and direct measurements such as corneal hysteresis may more accurately reflect ocular structural susceptibility than isolated regional thickness measurements alone. Future studies incorporating these biomechanical parameters may help clarify the relationship between anterior segment structure and posterior segment neurodegeneration.

The strong association observed between glaucoma diagnosis and RNFL thickness must also be interpreted in light of the study design. Glaucoma classification and severity staging were based in part on OCT-derived structural parameters, including RNFL thickness. Therefore, the association between glaucoma status and RNFL thinning reflects both biological disease progression and structural classification criteria. While this approach is consistent with established clinical diagnostic standards, it may partially amplify the strength of this association and should be interpreted accordingly.

Treatment-related factors also warrant consideration. All glaucoma patients were receiving stable antiglaucoma therapy for at least 12 months prior to evaluation, minimizing variability related to early treatment effects. However, chronic exposure to topical medications, particularly prostaglandin analogs, has been associated with gradual corneal thinning [42,43]. Although disease severity was included as a covariate to partially account for cumulative treatment exposure, detailed longitudinal treatment duration and medication-specific effects were not available. Therefore, treatment-related structural changes cannot be completely excluded as contributing factors.

Intraocular pressure was measured using non-contact tonometry in order to avoid potential corneal surface disruption associated with contact-based methods. While Goldmann applanation tonometry remains the clinical reference standard, non-contact tonometry also provides reliable measurements and avoids direct mechanical contact with the cornea. Importantly, the primary objective of this study was not to evaluate intraocular pressure measurement accuracy but rather to assess structural relationships between corneal and retinal parameters. References to Goldmann applanation tonometry in the literature provide important context regarding the biomechanical relevance of corneal thickness but do not directly affect the structural associations analyzed in this study.

The relationship between corneal thickness and optic nerve head structure remains complex and incompletely understood. Previous studies have reported inconsistent findings, with some demonstrating associations between thinner corneas and greater glaucomatous damage, while others found no significant relationship [44,45,46,47,48,49,50,51,52,53,54]. These discrepancies may reflect differences in study populations, disease severity, measurement techniques, and biomechanical characteristics.

Our findings align with studies demonstrating that corneal thickness is more strongly associated with glaucoma susceptibility than with the extent of existing neurodegeneration. This supports the concept that corneal thickness reflects intrinsic biomechanical vulnerability rather than serving as a marker of structural damage severity.

The correlation observed between RNFL thickness and rim area further supports the validity of the structural measurements used in this study. These parameters reflect complementary aspects of optic nerve head integrity and have been consistently shown to correlate with glaucoma progression [30,55,56].

Although ganglion cell-inner plexiform layer (GCIPL) analysis was not included in this study, it represents an additional structural biomarker of glaucomatous damage. GCIPL measurements may be particularly useful in early disease detection and longitudinal monitoring. Future studies incorporating both RNFL and GCIPL parameters may provide a more comprehensive assessment of neurodegeneration.

This study has several limitations that should be considered when interpreting the findings.

First, the cross-sectional design precludes causal inference and does not allow determination of whether reduced corneal thickness precedes the development of glaucomatous damage or arises as a consequence of disease progression or long-term treatment. Longitudinal studies are required to clarify the temporal relationship between corneal structural characteristics and neuroretinal degeneration.

Second, the glaucoma group was significantly older than the control group, reflecting the well-established age-related increase in glaucoma prevalence. Although age was included as a continuous covariate in all multivariable generalized estimating equation models and did not demonstrate an independent association with RNFL thickness after adjustment, residual confounding cannot be entirely excluded. Aging is associated with progressive RNFL thinning, extracellular matrix remodeling, and structural alterations in both retinal and corneal tissues, including changes in collagen organization, endothelial function, and biomechanical properties. These age-related processes may partially contribute to the observed structural differences despite statistical adjustment.

Third, treatment-related factors represent an additional potential source of confounding. All glaucoma patients were receiving chronic topical therapy, and long-term use of antiglaucoma medications—particularly prostaglandin analogs—has been associated with gradual corneal thinning and extracellular matrix remodeling. Although inclusion criteria required stable treatment for at least 12 months and disease severity was incorporated as a covariate to partially account for cumulative disease and treatment burden, detailed information regarding medication class, treatment duration, and cumulative exposure was not consistently available. Consequently, medication-induced structural changes cannot be completely excluded as contributors to the observed corneal thickness differences.

Fourth, intraocular pressure was measured using non-contact tonometry rather than Goldmann applanation tonometry. While non-contact tonometry avoids corneal contact and potential epithelial disruption, it may be more influenced by corneal thickness and biomechanical properties than applanation-based methods. Furthermore, longitudinal intraocular pressure data and cumulative pressure exposure were not incorporated into statistical models. Because intraocular pressure represents the primary modifiable risk factor for glaucoma progression, the absence of detailed pressure-related parameters limits mechanistic interpretation of the relationship between corneal structure and neuroretinal damage.

Fifth, glaucoma classification and disease severity staging were based in part on OCT-derived structural parameters, including RNFL thickness, which was also analyzed as an outcome variable. This methodological overlap reflects standard clinical diagnostic practice but may partially amplify the strength of association between glaucoma status and RNFL thickness. Therefore, these associations should be interpreted primarily as confirmation of disease-related structural differences rather than independent mechanistic relationships.

Sixth, biomechanical parameters such as corneal hysteresis, corneal resistance factor, scleral stiffness, and lamina cribrosa morphology were not assessed. These parameters may provide more direct insight into tissue susceptibility to glaucomatous damage than corneal thickness alone. Corneal thickness represents only one component of a broader biomechanical system, and the absence of complementary biomechanical measurements limits comprehensive characterization of ocular structural vulnerability.

Seventh, although sectoral corneal thickness was analyzed to explore potential regional associations with RNFL thickness, these analyses were exploratory in nature. Most associations did not remain statistically significant after correction for multiple testing, and therefore no definitive sector-specific relationships can be established. Larger studies incorporating biomechanical measurements and greater statistical power are needed to further evaluate potential regional susceptibility patterns.

Eighth, GCIPL parameters were not included in the analysis. GCIPL measurements may provide complementary information regarding glaucomatous neurodegeneration, particularly in early disease stages, and their inclusion could have strengthened structural characterization of optic nerve damage.

Ninth, the study population was recruited from a tertiary referral center, and more than half of glaucoma eyes were classified as severe. This may limit generalizability to broader glaucoma populations, particularly patients with early-stage or untreated disease.

Tenth, another potential limitation of the present study is the absence of axial length measurements in the multivariable analysis. Axial length and refractive status, particularly myopia, are known to influence both retinal nerve fiber layer thickness and susceptibility to glaucoma, and may also affect corneal structural parameters. Previous studies have demonstrated that eyes with increased axial length may exhibit thinner RNFL measurements and structural alterations of the optic nerve head that could confound glaucoma assessment. In the current study, eyes with high myopia associated with myopic retinal atrophy or degenerative changes were excluded in order to minimize the impact of extreme axial elongation on RNFL measurements and optic nerve morphology. However, axial length measurements were not consistently available for all participants and therefore could not be included as a continuous covariate in the multivariable models. Consequently, residual confounding related to axial length cannot be completely excluded. Future studies incorporating systematic axial length measurements and detailed refractive status may further clarify the relationship between corneal thickness, ocular biomechanics, and glaucomatous neurodegeneration.

Finally, although statistical models adjusted for multiple demographic and anatomical covariates and accounted for inter-eye correlation, unmeasured confounding factors—including systemic vascular conditions, genetic susceptibility, and environmental influences—cannot be completely excluded.

Despite these limitations, this study provides novel insights into the structural relationship between corneal thickness and glaucomatous neurodegeneration using detailed sectoral OCT-based analysis. The findings support the concept that corneal thickness reflects biomechanical susceptibility rather than serving as a direct indicator of neurodegenerative severity.

Future longitudinal studies incorporating biomechanical measurements, treatment exposure data, intraocular pressure dynamics, and multimodal structural parameters—including GCIPL and lamina cribrosa imaging—will be essential to further elucidate the role of corneal structure in glaucoma pathophysiology and improve risk stratification.

## 5. Conclusions

This study demonstrates that corneal thickness is significantly reduced in eyes with primary open-angle glaucoma compared with healthy controls; however, neither global nor sectoral corneal thickness showed independent associations with retinal nerve fiber layer thickness or optic disc rim area after adjustment for disease status and relevant confounders. These findings indicate that corneal thinning reflects a structural and biomechanical susceptibility factor rather than a direct marker of the extent or severity of glaucomatous neurodegeneration.

Importantly, exploratory sectoral analyses did not reveal consistent region-specific relationships between corneal thickness and RNFL parameters after correction for multiple testing, suggesting that localized corneal thickness variations alone are unlikely to serve as reliable indicators of sectoral neuroretinal damage.

Taken together, these results support the role of corneal thickness as a component of the broader biomechanical phenotype associated with glaucoma risk, rather than as a surrogate marker of established structural damage. The absence of independent associations between corneal thickness and RNFL thickness underscores the importance of direct structural imaging for assessing disease severity.

Future prospective studies incorporating longitudinal follow-up, detailed treatment exposure data, intraocular pressure dynamics, and comprehensive biomechanical assessments—including corneal hysteresis, corneal resistance factor, and lamina cribrosa imaging—are needed to further elucidate the relationship between anterior segment biomechanics and glaucomatous neurodegeneration, and to determine whether biomechanical parameters may improve risk stratification and early detection.

## Figures and Tables

**Table 1:** Demographic characteristics of the study participants: overall and stratified by group (glaucoma vs. control).

Characteristic	Overall (N = 192)	Glaucoma (N = 91)	Control (N = 101)	*p* ^1^
Age (years) ^2^	70.0 (56.0, 75.0) CI: 64.0, 69.0 [22.0, 91.0]	72.0 (67.0, 78.0) CI: 70.0, 74.0 [28.0, 91.0]	66.0 (51.0, 72.0) CI: 58.0, 64.0 [22.0, 85.0]	<0.001
Sex ^3^				0.729
Female	108 (56.3%), CI: 49.0%, 63.0%	50 (55.0%), CI: 44.0%, 65.0%	58 (57.4%), CI: 47.0%, 67.0%	
Male	84 (43.8%), CI: 37.0%, 51.0%	41 (45.1%), CI: 35.0%, 56.0%	43 (42.6%), CI: 33.0%, 53.0%	

Notes: CI = Confidence Interval. ^1^ *p*-values derived from Wilcoxon rank sum test for continuous variables and Pearson’s Chi-squared test for categorical variables. ^2^ Reported as median (Q1, Q3), CI: lower limit, upper limit [minimum, maximum]. ^3^ Reported as n (%). Confidence intervals for continuous variables were calculated using the Wilcox. test method; for categorical variables, using the Wilson method.

**Table 2:** Repeated measures correlations between rim area and corneal thickness parameters by group.

Group	CT Region	Correlation (r)	df	*p*-Value	95% CI
Glaucoma	Superior	0.034	53	0.803	−0.200, 0.290
Glaucoma	Temporal	−0.264	53	0.051	−0.495, 0.001
Glaucoma	Inferior	−0.139	53	0.311	−0.390, 0.131
Glaucoma	Nasal	0.033	53	0.813	−0.235, 0.295
Glaucoma	Central	−0.137	53	0.318	−0.388, 0.133
Control	Superior	−0.154	50	0.277	−0.409, 0.125
Control	Temporal	0.138	50	0.331	−0.141, 0.396
Control	Inferior	0.109	50	0.443	−0.169, 0.371
Control	Nasal	−0.024	50	0.866	−0.295, 0.251
Control	Central	0.092	50	0.516	−0.186, 0.356

Notes: CT = Corneal Thickness; df = Degrees of Freedom; CI = Confidence Interval. Correlation coefficients (r) range from −1 to 1, with values closer to 0 indicating weaker associations. *p*-values are two-tailed; values < 0.05 indicate statistical significance at the 5% level. Confidence intervals were derived analytically. No adjustments for multiple testing were performed, as analyses were hypothesis-generating.

## Data Availability

The data that support the findings of this study are available from the corresponding author upon reasonable request.

## Supplementary Materials

The following supporting information can be downloaded at: https://www.mdpi.com/article/10.3390/jcm15062405/s1, Table S1. Shapiro–Wilk normality test results for ophthalmic measurements by group. Table S2. Specifications of dataset, outcome variables, and predictor variables for fitted generalized estimating equation (GEE) models. Table S3. GEE model 1—association between average retinal nerve fiber layer (RNFL) thickness and superior corneal thickness (CT) in the overall sample. Table S4. GEE model 2—association between average RNFL thickness and temporal CT in the overall sample. Table S5. GEE model 3—association between average RNFL thickness and inferior CT in the overall sample. Table S6. GEE model 4—association between average RNFL thickness and nasal CT in the overall sample. Table S7. GEE model 5—association between average RNFL thickness and central CT in the overall sample. Table S8. GEE model 6—association between superior RNFL thickness and superior CT in the overall sample. Table S9. GEE model 7—association between temporal RNFL thickness and temporal CT in the overall sample. Table S10. GEE model 8—association between inferior RNFL thickness and inferior CT in the overall sample. Table S11. GEE model 9—association between nasal RNFL thickness and nasal CT in the overall sample. Table S12. GEE model 10—association between average RNFL thickness and superior CT among glaucoma patients. Table S13. GEE model 11—association between average RNFL thickness and temporal CT among glaucoma patients. Table S14. GEE model 12—association between average RNFL thickness and inferior CT among glaucoma patients. Table S15. GEE model 13—association between average RNFL thickness and nasal CT among glaucoma patients. Table S16. GEE model 14—association between average RNFL thickness and central CT among glaucoma patients. Table S17. GEE model 15—association between superior RNFL thickness and superior CT among glaucoma patients. Table S18. GEE model 16—association between temporal RNFL thickness and temporal CT among glaucoma patients. Table S19. GEE model 17—association between inferior RNFL thickness and inferior CT among glaucoma patients. Table S20. GEE model 18—association between nasal RNFL thickness and nasal CT among glaucoma patients. Figure S1: Scatterplots illustrating the relationship between average RNFL thickness and regional corneal thickness in glaucoma and control groups. Figure S2: Scatterplots illustrating the relationship between quadrant-matched RNFL thickness and corresponding corneal thickness in glaucoma and control groups.

## Abbreviations

The following abbreviations are used in this manuscript:

AGIS	Advanced Glaucoma Intervention Study
AS-OCT	Anterior Segment Optical Coherence Tomography
BCVA	Best-Corrected (Distance) Visual Acuity
CCT	Central Corneal Thickness
CH	Corneal Hysteresis
CI	Confidence Interval
CRF	Corneal Resistance Factor
CT	Corneal Thickness (sectoral; superior/temporal/inferior/nasal/central)
df	Degrees of Freedom
FDR	False Discovery Rate
GAT	Goldmann Applanation Tonometry
GCC	Ganglion Cell Complex
GEE	Generalized Estimating Equations
IOP	Intraocular Pressure
IQR	Interquartile Range
ISNT rule	Inferior > Superior > Nasal > Temporal (neuroretinal rim/RNFL pattern)
IST rule	Inferior > Superior > Temporal (simplified ISNT variant)
MD	Mean Deviation (visual field)
NCT	Non-Contact Tonometry
NRR	Neuroretinal Rim
OCT	Optical Coherence Tomography
OHT	Ocular Hypertension
OHTS	Ocular Hypertension Treatment Study
ONH	Optic Nerve Head
PADJ/*p*_adj_	FDR-adjusted *p*-value
POAG	Primary Open-Angle Glaucoma
PSD	Pattern Standard Deviation (visual field)
RA	Rim Area (optic disc)
RNFL	Retinal Nerve Fiber Layer
SD-OCT	Spectral-Domain Optical Coherence Tomography
VF	Visual Field
β	Regression coefficient (GEE)
*p*	Two-sided *p*-value
*r*	Correlation coefficient
μm	Micrometer
mm^2^	Square millimeter
mmHg	Millimeters of mercury

## Appendix A

**Table A1:** Ophthalmological characteristics of the study participants at the eye level: overall and stratified by group (glaucoma vs. control).

Characteristic	Overall (N = 297)	Glaucoma (N = 145)	Control (N = 152)	*p* ^1^
Eye				0.456
OD (Right Eye)	152 (51.2%) CI: 45.0%, 57.0%	71 (49.0%) CI: 41.0%, 57.0%	81 (53.3%) CI: 45.0%, 61.0%	
OS (Left Eye)	145 (48.8%) CI: 43.0%, 55.0%	74 (51.0%) CI: 43.0%, 59.0%	71 (46.7%) CI: 39.0%, 55.0%	
Optic disc parameters				
Rim area (mm^2^) ^2^	1.3 (0.8, 1.6) CI: 1.2, 1.3 [0.0, 2.7]	0.8 (0.3, 1.0) CI: 0.7, 0.9 [0.0, 2.7]	1.5 (1.4, 1.8) CI: 1.5, 1.6 [0.7, 2.7]	<0.001
Retinal nerve fiber layer thickness (μm)				
Average RNFL thickness ^2^	81.0 (63.0, 94.0) CI: 76.0, 81.0 [34.0, 121.0]	62.0 (51.0, 69.0) CI: 58.0, 63.0 [34.0, 82.0]	93.5 (87.0, 101.0) CI: 93.0, 96.0 [75.0, 121.0]	<0.001
Superior RNFL thickness ^2^	95.0 (66.0, 114.0) CI: 87.0, 95.0 [32.0, 156.0]	66.0 (49.0, 81.0) CI: 62.0, 69.0 [32.0, 104.0]	113.0 (105.0, 123.0) CI: 112.0, 116.0 [86.0, 156.0]	<0.001
Temporal RNFL thickness ^2^	59.0 (47.0, 66.0) CI: 56.0, 60.0 [31.0, 126.0]	47.0 (38.0, 59.0) CI: 47.0, 52.0 [31.0, 126.0]	64.5 (59.0, 69.0) CI: 63.0, 66.0 [48.0, 114.0]	<0.001
Inferior RNFL thickness ^2^	102.0 (67.0, 122.0) CI: 91.0, 100.0 [32.0, 175.0]	64.0 (46.0, 86.0) CI: 63.0, 70.0 [32.0, 114.0]	121.5 (112.5, 133.5) CI: 120.0, 125.0 [92.0, 175.0]	<0.001
Nasal RNFL thickness ^2^	71.0 (58.0, 82.0) CI: 68.0, 72.0 [32.0, 132.0]	57.0 (50.0, 66.0) CI: 56.0, 60.0 [32.0, 107.0]	80.5 (73.0, 89.0) CI: 79.0, 83.0 [60.0, 132.0]	<0.001
Corneal thickness (μm)				
Superior corneal thickness ^2^	629.0 (592.0, 668.0) CI: 625.0, 637.0 [516.0, 836.0]	607.0 (581.0, 653.0) CI: 610.0, 628.0 [516.0, 757.0]	640.0 (613.0, 673.0) CI: 634.0, 650.0 [522.0, 836.0]	<0.001
Temporal corneal thickness ^2^	572.0 (547.0, 603.0) CI: 570.0, 581.0 [436.0, 728.0]	562.0 (540.0, 596.0) CI: 558.0, 573.0 [436.0, 690.0]	579.5 (557.0, 611.5) CI: 578.0, 592.0 [440.0, 728.0]	<0.001
Inferior corneal thickness ^2^	589.0 (563.0, 620.0) CI: 585.0, 595.0 [402.0, 772.0]	582.0 (554.0, 622.0) CI: 577.0, 592.0 [402.0, 737.0]	592.0 (571.0, 618.0) CI: 588.0, 601.0 [438.0, 772.0]	0.027
Nasal corneal thickness ^2^	599.0 (575.0, 631.0) CI: 597.0, 607.0 [424.0, 757.0]	588.0 (562.0, 621.0) CI: 585.0, 600.0 [424.0, 757.0]	610.5 (584.5, 636.5) CI: 604.0, 617.0 [431.0, 720.0]	<0.001
Central corneal thickness ^2^	537.0 (512.0, 563.0) CI: 533.0, 542.0 [379.0, 640.0]	527.0 (508.0, 555.0) CI: 524.0, 537.0 [390.0, 640.0]	543.5 (518.0, 570.0) CI: 538.0, 550.0 [379.0, 640.0]	0.003
Glaucoma severity				
Severe glaucoma ^3^	81 (55.9%) ^4^ CI: 47.0%, 64.0%	81 (55.9%) CI: 47.0%, 64.0%	Not applied	-

Abbreviations: CI = Confidence Interval; RNFL = Retinal Nerve Fiber Layer; OD = Oculus Dexter; OS = Oculus Sinister. ^1^ *p*-values derived from Wilcoxon rank sum test for continuous variables, Pearson’s Chi-squared test for categorical variables, and Fisher’s exact test where appropriate. ^2^ Reported as median (Q1, Q3), CI: lower limit, upper limit [minimum, maximum]. ^3^ Reported as n (%); CI: lower limit, upper limit. ^4^ For Severe Glaucoma in the overall sample, N = 145 (applicable only to glaucoma eyes). Confidence intervals for continuous variables were calculated using the Wilcox test method; for categorical variables, using the Wilson method.
