# Supplementary material for: Sectoral Analysis of Corneal Thickness in Glaucoma and Healthy Eyes and Its Relationship with RNFL and Rim Area
Source: J Clin Med. 2026 Mar 21;15(6):2405. doi: 10.3390/jcm15062405 (PMC13027029; doi:10.3390/jcm15062405)
Supplement: Supplementary file 1 [file jcm-15-02405-s001.zip › jcm-4153825-supplementary.pdf]

Supplementary

Table S.1. Shapiro-Wilk normality test results for ophthalmic measurements by group

Parameter	Group	n	Shapiro-Wilk Statistic	p
Average RNFL Thickness	Control	152	0.982	**0.039**
	Glaucoma	145	0.952	< **0.001**
Superior RNFL Thickness	Control	152	0.984	0.073
	Glaucoma	145	0.961	< **0.001**
Temporal RNFL Thickness	Control	152	0.870	< **0.001**
	Glaucoma	145	0.898	< **0.001**
Inferior RNFL Thickness	Control	152	0.979	**0.021**
	Glaucoma	145	0.944	< **0.001**
Nasal RNFL Thickness	Control	152	0.956	< **0.001**
	Glaucoma	145	0.952	< **0.001**
Superior CT	Control	152	0.978	**0.016**
	Glaucoma	145	0.973	**0.005**
Temporal CT	Control	152	0.980	**0.026**
	Glaucoma	145	0.991	0.470
Inferior CT	Control	152	0.949	< **0.001**
	Glaucoma	145	0.981	**0.042**
Nasal CT	Control	152	0.971	**0.003**
	Glaucoma	145	0.977	**0.014**
Central CT	Control	152	0.973	**0.004**
	Glaucoma	145	0.989	0.339

Notes: RNFL = Retinal Nerve Fiber Layer; CT = Corneal Thickness. Notes: n represents the eye-level sample size for each group. The Shapiro-Wilk statistic ranges from 0 to 1, with values closer to 1 indicating greater conformity to normality. p-values below 0.05 indicate rejection of the null hypothesis of normality, demonstrating that the data distribution deviates significantly from normal.

Table S.2. Specifications of dataset, outcome variables, and predictor variables for fitted generalized estimating equation models

Model id	Data	Outcome	Predictor
Model 1	Overall sample	Average RNFL thickness	Superior corneal thickness
Model 2			Temporal corneal thickness
Model 3			Inferior corneal thickness
Model 4			Nasal corneal thickness
Model 5			Central corneal thickness
Model 6		Superior RNFL thickness	Superior corneal thickness
Model 7		Temporal RNFL thickness	Temporal corneal thickness
Model 8		Inferior RNFL thickness	Inferior corneal thickness
Model 9		Nasal RNFL thickness	Nasal corneal thickness
Model 10	Glaucoma group	Average RNFL thickness	Superior corneal thickness
Model 11			Temporal corneal thickness
Model 12			Inferior corneal thickness
Model 13			Nasal corneal thickness
Model 14			Central corneal thickness
Model 15		Superior RNFL thickness	Superior corneal thickness
Model 16		Temporal RNFL thickness	Temporal corneal thickness
Model 17		Inferior RNFL thickness	Inferior corneal thickness
Model 18		Nasal RNFL thickness	Nasal corneal thickness

Note: RNFL denotes Retinal Nerve Fiber Layer. Models 1–9 were fitted to the overall study sample, incorporating an interaction term for group (glaucoma vs. control). Models 10–18 were restricted to the glaucoma subgroup, excluding the group interaction. All models adjusted for age, sex, eye laterality, and rim area as covariates.

Table S.3. Generalized estimating equation *model 1* results – association between *average retinal nerve fiber layer thickness* and *superior corneal thickness* in the overall sample

**Parameter**	**β**	**SE**	**95% CI**	**z**	**p**	**p_{adj}**
Intercept	92.30	1.20	(89.96, 94.65)	77.23	**< 0.001**	**< 0.001**
Superior CT (centered at 629 µm)	0.01	0.01	(-0.01, 0.03)	0.54	0.591	0.874
Group (Glaucoma vs. Control)	-27.83	1.58	(-30.93, -24.72)	-17.56	**< 0.001**	**< 0.001**
Age (centered at 70 years)	-0.05	0.05	(-0.15, 0.05)	-1.01	0.311	0.794
Sex (Male vs. Female)	-0.13	1.33	(-2.73, 2.47)	-0.10	0.923	0.923
Rim Area (centered at 1.3 mm²)	7.68	1.32	(5.09, 10.26)	5.81	**< 0.001**	**< 0.001**
Eye (OS vs. OD)	0.19	0.71	(-1.21, 1.59)	0.27	0.786	0.881
Superior CT (centered at 629) × Group (Glaucoma vs. Control)	0.04	0.02	(-0.00, 0.08)	1.88	0.060	0.135

Notes: SE = Standard Error; CI = Confidence Interval; RNFL = Retinal Nerve Fiber Layer; CT = Corneal Thickness; OD = Oculus Dexter (Right Eye); OS = Oculus Sinister (Left Eye). The outcome variable is *Average RNFL thickness* (in µm). Model *1* was fitted to the overall sample using a GEE approach with an exchangeable working correlation structure to account for within-patient clustering of bilateral eye measurements. Continuous predictors (*Superior* CT, Age, Rim Area) were centered at their respective medians for interpretability. The model adjusts for age, sex, rim area, and eye laterality as covariates. Coefficients represent population-averaged effects; p-values are based on robust standard errors. p_{adj} represents FDR-adjusted p-values using the Benjamini-Hochberg method across the models 1-9 for each parameter type. A p-value < 0.05 indicates statistical significance at the 5% level.

Table S.4. Generalized estimating equation *model 2* results – association between *average retinal nerve fiber layer thickness* and *temporal corneal thickness* in the overall sample

**Parameter**	**β**	**SE**	**95% CI**	**z**	**p**	**p_{adj}**
Intercept	92.52	1.25	(90.07, 94.97)	74.02	**< 0.001**	**< 0.001**
Temporal CT (centered at 572 µm)	-0.01	0.02	(-0.04, 0.02)	-0.48	0.628	0.874
Group (Glaucoma vs. Control)	-28.21	1.64	(-31.42, -25.00)	-17.22	**< 0.001**	**< 0.001**
Age (centered at 70 years)	-0.04	0.05	(-0.14, 0.06)	-0.83	0.404	0.794
Sex (Male vs. Female)	-0.23	1.32	(-2.81, 2.36)	-0.17	0.863	0.923
Rim Area (centered at 1.3 mm²)	7.67	1.35	(5.02, 10.32)	5.68	**< 0.001**	**< 0.001**
Eye (OS vs. OD)	0.39	0.71	(-1.00, 1.79)	0.55	0.582	0.881
Temporal CT (centered at 572 µm) × Group (Glaucoma vs. Control)	0.05	0.03	(0.00, 0.11)	2.03	**0.042**	0.126

Notes: SE = Standard Error; CI = Confidence Interval; RNFL = Retinal Nerve Fiber Layer; CT = Corneal Thickness; OD = Oculus Dexter (Right Eye); OS = Oculus Sinister (Left Eye). The outcome variable is *Average RNFL thickness* (in µm). Model *2* was fitted to the overall sample using a GEE approach with an exchangeable working correlation structure to account for within-patient clustering of bilateral eye measurements. Continuous predictors (*Temporal* CT, Age, Rim Area) were centered at their respective medians for interpretability. The model adjusts for age, sex, rim area, and eye laterality as covariates. Coefficients represent population-averaged effects; p-values are based on robust standard errors. p_{adj} represents FDR-adjusted p-values using the Benjamini-Hochberg method across the models 1-9 for each parameter type. A p-value < 0.05 indicates statistical significance at the 5% level.

Table S.5. Generalized estimating equation *model 3* results – association *between average retinal nerve fiber layer thickness* and *inferior corneal thickness* in the overall sample

Parameter	β	SE	95% CI	z	p	p_{adj}
Intercept	92.60	1.21	(90.22, 94.97)	76.37	**< 0.001**	**< 0.001**
Inferior CT (centered at 589 µm)	-0.01	0.01	(-0.04, 0.02)	-0.80	0.425	0.874
Group (Glaucoma vs. Control)	-28.34	1.63	(-31.54, -25.15)	-17.40	**< 0.001**	**< 0.001**
Age (centered at 70 years)	-0.04	0.05	(-0.13, 0.05)	-0.87	0.382	0.794
Sex (Male vs. Female)	-0.20	1.32	(-2.78, 2.39)	-0.15	0.881	0.923
Rim Area (centered at 1.3 mm²)	7.49	1.35	(4.84, 10.14)	5.55	**< 0.001**	**< 0.001**
Eye (OS vs. OD)	0.27	0.71	(-1.11, 1.66)	0.39	0.698	0.881
Inferior CT (centered at 589 µm) × Group (Glaucoma vs. Control)	0.06	0.03	(0.01, 0.11)	2.40	**0.016**	0.074

Notes: SE = Standard Error; CI = Confidence Interval; RNFL = Retinal Nerve Fiber Layer; CT = Corneal Thickness; OD = Oculus Dexter (Right Eye); OS = Oculus Sinister (Left Eye). The outcome variable is *Average RNFL thickness* (in µm). Model *3* was fitted to the overall sample using a GEE approach with an exchangeable working correlation structure to account for within-patient clustering of bilateral eye measurements. Continuous predictors (*Inferior* CT, Age, Rim Area) were centered at their respective medians for interpretability. The model adjusts for age, sex, rim area, and eye laterality as covariates. Coefficients represent population-averaged effects; p-values are based on robust standard errors p_{adj} represents FDR-adjusted p-values using the Benjamini-Hochberg method across the models 1-9 for each parameter type. A p-value < 0.05 indicates statistical significance at the 5% level.

Table S.6. Generalized estimating equation model 4 results – association between *average retinal nerve fiber layer thickness* and *nasal corneal thickness* in the overall sample

Parameter	β	SE	95% CI	z	p	p_{adj}
Intercept	92.35	1.18	(90.04, 94.67)	78.22	**< 0.001**	**< 0.001**
Nasal CT (centered at 599 µm)	0.00	0.02	(-0.03, 0.03)	0.16	0.874	0.874
Group (Glaucoma vs. Control)	-27.98	1.61	(-31.13, -24.83)	-17.43	**< 0.001**	**< 0.001**
Age (centered at 70 years)	-0.05	0.05	(-0.14, 0.04)	-1.05	0.293	0.794
Sex (Male vs. Female)	-0.21	1.33	(-2.83, 2.40)	-0.16	0.874	0.923
Rim Area (centered at 1.3 mm²)	7.77	1.35	(5.12, 10.43)	5.75	**< 0.001**	**< 0.001**
Eye (OS vs. OD)	0.19	0.71	(-1.21, 1.60)	0.27	0.786	0.881
Nasal CT (centered at 599 µm) × Group (Glaucoma vs. Control)	0.03	0.02	(-0.02, 0.07)	1.07	0.285	0.365

Notes: SE = Standard Error; CI = Confidence Interval; RNFL = Retinal Nerve Fiber Layer; CT = Corneal Thickness; OD = Oculus Dexter (Right Eye); OS = Oculus Sinister (Left Eye). The outcome variable is *Average RNFL thickness* (in µm). Model *4* was fitted to the overall sample using a GEE approach with an exchangeable working correlation structure to account for within-patient clustering of bilateral eye measurements. Continuous predictors (*Nasal* CT, Age, Rim Area) were centered at their respective medians for interpretability. The model adjusts for age, sex, rim area, and eye laterality as covariates. Coefficients represent population-averaged effects; p-values are based on robust standard errors. p_{adj} represents FDR-adjusted p-values using the Benjamini-Hochberg method across the models 1-9 for each parameter type. A p-value < 0.05 indicates statistical significance at the 5% level.

Table S.7. Generalized estimating equation model 5 results – association between *average retinal nerve fiber layer thickness* and *central corneal thickness* in the overall sample

Parameter	β	SE	95% CI	z	p	p_{adj}
Intercept	92.47	1.20	(90.12, 94.82)	77.14	**< 0.001**	**< 0.001**
Central CT (centered at 537 µm)	0.01	0.02	(-0.02, 0.04)	0.55	0.581	0.874
Group (Glaucoma vs. Control)	-28.12	1.60	(-31.26, -24.98)	-17.54	**< 0.001**	**< 0.001**
Age (centered at 70 years)	-0.04	0.05	(-0.13, 0.06)	-0.81	0.418	0.794
Sex (Male vs. Female)	-0.31	1.32	(-2.90, 2.28)	-0.23	0.816	0.923
Rim Area (centered at 1.3 mm²)	7.46	1.31	(4.88, 10.03)	5.67	**< 0.001**	**< 0.001**
Eye (OS vs. OD)	0.36	0.71	(-1.03, 1.75)	0.50	0.614	0.881
Central CT (centered at 537 µm) × Group (Glaucoma vs. Control)	0.05	0.03	(-0.01, 0.11)	1.67	0.096	0.171

Notes: SE = Standard Error; CI = Confidence Interval; RNFL = Retinal Nerve Fiber Layer; CT = Corneal Thickness; OD = Oculus Dexter (Right Eye); OS = Oculus Sinister (Left Eye). The outcome variable is *Average RNFL thickness* (in µm). Model *5* was fitted to the overall sample using a GEE approach with an exchangeable working correlation structure to account for within-patient clustering of bilateral eye measurements. Continuous predictors (*Central* CT, Age, Rim Area) were centered at their respective medians for interpretability. The model adjusts for age, sex, rim area, and eye laterality as covariates. Coefficients represent population-averaged effects; p-values are based on robust standard errors. p_{adj} represents FDR-adjusted p-values using the Benjamini-Hochberg method across the models 1-9 for each parameter type.A p-value < 0.05 indicates statistical significance at the 5% level.

Table S.8. Generalized estimating equation model 6 results – association between *superior retinal nerve fiber layer thickness* and *superior corneal thickness* in the overall sample

Parameter	β	SE	95% CI	z	p	p_{adj}
Intercept	109.25	1.87	(105.58, 112.92)	58.38	**< 0.001**	**< 0.001**
Superior CT (centered at 629 µm)	0.02	0.02	(-0.02, 0.06)	1.05	0.293	0.874
Group (Glaucoma vs. Control)	-37.31	2.80	(-42.80, -31.81)	-13.31	**< 0.001**	**< 0.001**
Age (centered at 70 years)	-0.02	0.07	(-0.16, 0.11)	-0.32	0.752	0.794
Sex (Male vs. Female)	0.43	1.97	(-3.44, 4.29)	0.22	0.829	0.923
Rim Area (centered at 1.3 mm²)	13.45	2.26	(9.03, 17.87)	5.97	**< 0.001**	**< 0.001**
Eye (OS vs. OD)	1.64	1.25	(-0.81, 4.09)	1.31	0.190	0.881
Superior CT (centered at 629 µm) × Group (Glaucoma vs. Control)	0.04	0.03	(-0.03, 0.10)	1.12	0.264	0.365

Notes: SE = Standard Error; CI = Confidence Interval; RNFL = Retinal Nerve Fiber Layer; CT = Corneal Thickness; OD = Oculus Dexter (Right Eye); OS = Oculus Sinister (Left Eye). The outcome variable is *Superior RNFL thickness* (in µm). Model *6* was fitted to the overall sample using a GEE approach with an exchangeable working correlation structure to account for within-patient clustering of bilateral eye measurements. Continuous predictors (*Superior* CT, Age, Rim Area) were centered at their respective medians for interpretability. The model adjusts for age, sex, rim area, and eye laterality as covariates. Coefficients represent population-averaged effects; p-values are based on robust standard errors. p_{adj} represents FDR-adjusted p-values using the Benjamini-Hochberg method across the models 1-9 for each parameter type. A p-value < 0.05 indicates statistical significance at the 5% level.

Table S.9. Generalized estimating equation model 7 results – association between *temporal retinal nerve fiber layer thickness* and *temporal corneal thickness* in the overall sample

Parameter	β	SE	95% CI	z	p	p_{adj}
Intercept	64.79	1.49	(61.87, 67.70)	43.53	**< 0.001**	**< 0.001**
Temporal CT (centered at 572 μm)	0.03	0.02	(-0.01, 0.07)	1.51	0.131	0.736
Group (Glaucoma vs. Control)	-11.00	2.46	(-15.83, -6.18)	-4.47	**< 0.001**	**< 0.001**
Age (centered at 70 years)	0.02	0.07	(-0.12, 0.15)	0.22	0.824	0.824
Sex (Male vs. Female)	-0.98	1.75	(-4.41, 2.45)	-0.56	0.575	0.874
Rim Area (centered at 1.3 mm²)	4.53	1.81	(0.97, 8.08)	2.50	**0.013**	**0.014**
Eye (OS vs. OD)	-0.17	1.14	(-2.41, 2.07)	-0.15	0.881	0.881
Temporal CT (centered at 572 μm) × Group (Glaucoma vs. Control)	-0.02	0.03	(-0.09, 0.04)	-0.74	0.461	0.517

Notes: SE = Standard Error; CI = Confidence Interval; RNFL = Retinal Nerve Fiber Layer; CT = Corneal Thickness; OD = Oculus Dexter (Right Eye); OS = Oculus Sinister (Left Eye). The outcome variable is *Temporal RNFL thickness* (in μm). Model *7* was fitted to the overall sample using a GEE approach with an exchangeable working correlation structure to account for within-patient clustering of bilateral eye measurements. Continuous predictors (*Temporal* CT, Age, Rim Area) were centered at their respective medians for interpretability. The model adjusts for age, sex, rim area, and eye laterality as covariates. Coefficients represent population-averaged effects; p-values are based on robust standard errors. p_{adj} represents FDR-adjusted p-values using the Benjamini-Hochberg method across the models 1-9 for each parameter type. A p-value < 0.05 indicates statistical significance at the 5% level.

Table S.10. Generalized estimating equation model 8 results – association between *inferior retinal nerve fiber layer thickness* and *inferior corneal thickness* in the overall sample

Parameter	β	SE	95% CI	z	p	p_{adj}
Intercept	118.08	2.03	(114.11, 122.06)	58.24	**< 0.001**	**< 0.001**
Inferior CT (centered at 589 μm)	-0.01	0.03	(-0.06, 0.04)	-0.34	0.736	0.874
Group (Glaucoma vs. Control)	-42.18	3.22	(-48.49, -35.87)	-13.11	**< 0.001**	**< 0.001**
Age (centered at 70 years)	-0.15	0.09	(-0.32, 0.02)	-1.75	0.080	0.794
Sex (Male vs. Female)	-3.05	2.16	(-7.29, 1.19)	-1.41	0.158	0.874
Rim Area (centered at 1.3 mm²)	12.38	2.79	(6.91, 17.85)	4.43	**< 0.001**	**< 0.001**
Eye (OS vs. OD)	2.10	1.56	(-0.96, 5.15)	1.34	0.179	0.881
Inferior CT (centered at 589 μm) × Group (Glaucoma vs. Control)	0.12	0.05	(0.03, 0.21)	2.64	**0.008**	0.074

Notes: SE = Standard Error; CI = Confidence Interval; RNFL = Retinal Nerve Fiber Layer; CT = Corneal Thickness; OD = Oculus Dexter (Right Eye); OS = Oculus Sinister (Left Eye). The outcome variable is *Inferior RNFL thickness* (in μm). Model *8* was fitted to the overall sample using a GEE approach with an exchangeable working correlation structure to account for within-patient clustering of bilateral eye measurements. Continuous predictors (*Inferior* CT, Age, Rim Area) were centered at their respective medians for interpretability. The model adjusts for age, sex, rim area, and eye laterality as covariates. Coefficients represent population-averaged effects; p-values are based on robust standard errors. p_{adj} represents FDR-adjusted p-values using the Benjamini-Hochberg method across the models 1-9 for each parameter type. A p-value < 0.05 indicates statistical significance at the 5% level.

Table S.11. Generalized estimating equation model 9 results – association between *nasal retinal nerve fiber layer thickness* and *nasal corneal thickness* in the overall sample

**Parameter**	**β**	**SE**	**95% CI**	**z**	**p**	**p_{adj}**
Intercept	80.79	1.70	(77.45, 84.13)	47.45	**< 0.001**	**< 0.001**
Nasal CT (centered at 599 μm)	-0.01	0.02	(-0.05, 0.03)	-0.53	0.593	0.874
Group (Glaucoma vs. Control)	-20.82	2.34	(-25.40, -16.24)	-8.91	**< 0.001**	**< 0.001**
Age (centered at 70 years)	-0.02	0.07	(-0.15, 0.12)	-0.26	0.794	0.794
Sex (Male vs. Female)	2.15	1.74	(-1.26, 5.56)	1.24	0.216	0.874
Rim Area (centered at 1.3 mm²)	3.38	2.14	(-0.81, 7.58)	1.58	0.114	0.114
Eye (OS vs. OD)	-1.49	1.01	(-3.48, 0.49)	-1.48	0.140	0.881
Nasal CT (centered at 599 μm) × Group (Glaucoma vs. Control)	0.00	0.03	(-0.06, 0.07)	0.14	0.886	0.886

Notes: SE = Standard Error; CI = Confidence Interval; RNFL = Retinal Nerve Fiber Layer; CT = Corneal Thickness; OD = Oculus Dexter (Right Eye); OS = Oculus Sinister (Left Eye). The outcome variable is *Nasal RNFL thickness* (in μm). Model *9* was fitted to the overall sample using a GEE approach with an exchangeable working correlation structure to account for within-patient clustering of bilateral eye measurements. Continuous predictors (*Nasal* CT, Age, Rim Area) were centered at their respective medians for interpretability. The model adjusts for age, sex, rim area, and eye laterality as covariates. Coefficients represent population-averaged effects; p-values are based on robust standard errors. p_{adj} represents FDR-adjusted p-values using the Benjamini-Hochberg method across the models 1-9 for each parameter type. A p-value < 0.05 indicates statistical significance at the 5% level.

Table S.12. Generalized estimating equation model 10 results – association between *average retinal nerve fiber layer thickness* and *superior corneal thickness* among glaucoma patients

**Parameter**	**β**	**SE**	**95% CI**	**z**	**p**	**p_{adj}**
Intercept	68.40	1.24	(65.96, 70.84)	54.95	**< 0.001**	**< 0.001**
Superior CT (centered at 607 μm)	0.02	0.01	(-0.01, 0.04)	1.33	0.183	0.915
Severe glaucoma (Yes vs. No)	-15.82	1.60	(-18.96, -12.68)	-9.87	**< 0.001**	**< 0.001**
Age (centered at 72 years)	-0.12	0.06	(-0.24, -0.00)	-2.00	**0.046**	0.153
Sex (Male vs. Female)	-0.56	1.51	(-3.51, 2.39)	-0.37	0.710	0.915
Rim Area (centered at 0.8 mm²)	2.56	1.51	(-0.39, 5.51)	1.70	0.090	0.360
Eye (OS vs. OD)	1.08	1.10	(-1.07, 3.24)	0.99	0.324	0.915
Superior CT (centered at 607 μm) × Severe glaucoma (Yes vs. No)	0.02	0.03	(-0.03, 0.07)	0.73	0.464	0.915

Notes: SE = Standard Error; CI = Confidence Interval; RNFL = Retinal Nerve Fiber Layer; CT = Corneal Thickness; OD = Oculus Dexter (Right Eye); OS = Oculus Sinister (Left Eye). The outcome variable is *Average RNFL thickness* (in μm). Model *10* was fitted to the glaucoma group using a GEE approach with an exchangeable working correlation structure to account for within-patient clustering of bilateral eye measurements. Continuous predictors (*Superior* CT, Age, Rim Area) were centered at their respective medians for interpretability. The model adjusts for glaucoma severity, age, sex, rim area, and eye laterality as covariates. Coefficients represent population-averaged effects; p-values are based on robust standard errors. p_{adj} represents FDR-adjusted p-values using the Benjamini-Hochberg method across the models 10-18 for each parameter type. A p-value < 0.05 indicates statistical significance at the 5% level.

Table S.13. Generalized estimating equation model 11 results – association between *average retinal nerve fiber layer thickness* and *temporal corneal thickness* among glaucoma patients

**Parameter**	**β**	**SE**	**95% CI**	**z**	**p**	$\mathbf{p_{adj}}$
Intercept	68.31	1.21	(65.94, 70.67)	55.55	**< 0.001**	**< 0.001**
Temporal CT (centered at 562 µm)	0.04	0.02	(0.00, 0.07)	2.03	**0.043**	0.153
Severe glaucoma (Yes vs. No)	-15.61	1.51	(-18.57, -12.64)	-10.31	**< 0.001**	**< 0.001**
Age (centered at 72 years)	-0.09	0.06	(-0.21, 0.03)	-1.52	0.128	0.576
Sex (Male vs. Female)	-0.70	1.54	(-3.72, 2.32)	-0.45	0.650	0.915
Rim Area (centered at 0.8 mm²)	2.39	1.50	(-0.54, 5.33)	1.60	0.110	0.360
Eye (OS vs. OD)	1.42	1.08	(-0.69, 3.54)	1.32	0.187	0.915
Temporal CT (centered at 562 µm) × Severe glaucoma (Yes vs. No)	0.00	0.03	(-0.06, 0.06)	0.05	0.960	0.960

Notes: SE = Standard Error; CI = Confidence Interval; RNFL = Retinal Nerve Fiber Layer; CT = Corneal Thickness; OD = Oculus Dexter (Right Eye); OS = Oculus Sinister (Left Eye). The outcome variable is *Average RNFL thickness* (in µm). Model *11* was fitted to the glaucoma group using a GEE approach with an exchangeable working correlation structure to account for within-patient clustering of bilateral eye measurements. Continuous predictors (*Temporal* CT, Age, Rim Area) were centered at their respective medians for interpretability. The model adjusts for glaucoma severity, age, sex, rim area, and eye laterality as covariates. Coefficients represent population-averaged effects; p-values are based on robust standard errors. p_{adj} represents FDR-adjusted p-values using the Benjamini-Hochberg method across the models 10-18 for each parameter type. A p-value < 0.05 indicates statistical significance at the 5% level.

Table S.14. Generalized estimating equation model 12 results – association between *average retinal nerve fiber layer thickness* and *inferior corneal thickness* among glaucoma patients

**Parameter**	**β**	**SE**	**95% CI**	**z**	**p**	$\mathbf{p_{adj}}$
Intercept	69.66	1.21	(66.29, 71.02)	56.93	**< 0.001**	**< 0.001**
Inferior CT (centered at 589 µm)	0.02	0.01	(-0.00, 0.05)	1.66	0.097	0.360
Severe glaucoma (Yes vs. No)	-15.39	1.57	(-18.47, -12.32)	-9.81	**< 0.001**	**< 0.001**
Age (centered at 72 years)	-0.10	0.06	(-0.22, 0.01)	-1.79	0.074	0.222
Sex (Male vs. Female)	-0.59	1.50	(-3.53, 2.36)	-0.39	0.696	0.915
Rim Area (centered at 0.8 mm²)	2.42	1.49	(-0.50, 5.34)	1.62	0.105	0.360
Eye (OS vs. OD)	1.14	1.07	(-0.96, 3.23)	1.06	0.288	0.915
Inferior CT (centered at 589 µm) × Severe glaucoma (Yes vs. No)	0.01	0.03	(-0.04, 0.07)	0.45	0.655	0.915

Notes: SE = Standard Error; CI = Confidence Interval; RNFL = Retinal Nerve Fiber Layer; CT = Corneal Thickness; OD = Oculus Dexter (Right Eye); OS = Oculus Sinister (Left Eye). The outcome variable is *Average RNFL thickness* (in µm). Model *12* was fitted to the glaucoma group using a GEE approach with an exchangeable working correlation structure to account for within-patient clustering of bilateral eye measurements. Continuous predictors (*Inferior* CT, Age, Rim Area) were centered at their respective medians for interpretability. The model adjusts for glaucoma severity, age, sex, rim area, and eye laterality as covariates. Coefficients represent population-averaged effects; p-values are based on robust standard errors. p_{adj} represents FDR-adjusted p-values using the Benjamini-Hochberg method across the models 10-18 for each parameter type. A p-value < 0.05 indicates statistical significance at the 5% level.

Table S.15. Generalized estimating equation model 13 results – association between *average retinal nerve fiber layer thickness* and *nasal corneal thickness* among glaucoma patients

Parameter	β	SE	95% CI	z	p	p_{adj}
Intercept	68.85	1.24	(66.42, 71.28)	55.57	**< 0.001**	**< 0.001**
Nasal CT (centered at 588 μm)	0.00	0.01	(-0.02, 0.03)	0.25	0.803	0.915
Severe glaucoma (Yes vs. No)	-15.88	1.59	(-18.99, -12.77)	-10.01	**< 0.001**	**< 0.001**
Age (centered at 72 years)	-0.13	0.06	(-0.25, -0.01)	-2.17	**0.030**	0.153
Sex (Male vs. Female)	-0.72	1.48	(-3.62, 2.18)	-0.49	0.627	0.915
Rim Area (centered at 0.8 mm²)	2.68	1.53	(-0.32, 5.67)	1.75	0.080	0.360
Eye (OS vs. OD)	0.99	1.09	(-1.15, 3.13)	0.91	0.363	0.915
Nasal CT (centered at 588 μm) × Severe glaucoma (Yes vs. No)	0.01	0.03	(-0.05, 0.07)	0.42	0.672	0.915

Notes: SE = Standard Error; CI = Confidence Interval; RNFL = Retinal Nerve Fiber Layer; CT = Corneal Thickness; OD = Oculus Dexter (Right Eye); OS = Oculus Sinister (Left Eye). The outcome variable is *Average RNFL thickness* (in μm). Model *13* was fitted to the glaucoma group using a GEE approach with an exchangeable working correlation structure to account for within-patient clustering of bilateral eye measurements. Continuous predictors (*Nasal* CT, Age, Rim Area) were centered at their respective medians for interpretability. The model adjusts for glaucoma severity, age, sex, rim area, and eye laterality as covariates. Coefficients represent population-averaged effects; p-values are based on robust standard errors. p_{adj} represents FDR-adjusted p-values using the Benjamini-Hochberg method across the models 10-18 for each parameter type. A p-value < 0.05 indicates statistical significance at the 5% level.

Table S.16. Generalized estimating equation model 14 results – association *between average retinal nerve fiber layer thickness* and *central corneal thickness* among glaucoma patients

Parameter	β	SE	95% CI	z	p	p_{adj}
Intercept	68.29	1.26	(65.81, 70.77)	54.05	**< 0.001**	**< 0.001**
Central CT (centered at 517 μm)	0.02	0.02	(-0.01, 0.06)	1.22	0.224	0.915
Severe glaucoma (Yes vs. No)	-15.73	1.62	(-18.90, -12.56)	-9.72	**< 0.001**	**< 0.001**
Age (centered at 72 years)	-0.11	0.06	(-0.23, 0.01)	-1.83	0.068	0.222
Sex (Male vs. Female)	-0.72	1.49	(-3.64, 2.21)	-0.48	0.631	0.915
Rim Area (centered at 0.8 mm²)	2.45	1.49	(-0.46, 5.36)	1.65	0.099	0.360
Eye (OS vs. OD)	1.17	1.08	(-0.95, 3.29)	1.08	0.279	0.915
Central CT (centered at 517 μm) × Severe glaucoma (Yes vs. No)	0.01	0.03	(-0.05, 0.08)	0.33	0.740	0.915

Notes: SE = Standard Error; CI = Confidence Interval; RNFL = Retinal Nerve Fiber Layer; CT = Corneal Thickness; OD = Oculus Dexter (Right Eye); OS = Oculus Sinister (Left Eye). The outcome variable is *Average RNFL thickness* (in μm). Model *14* was fitted to the glaucoma group using a GEE approach with an exchangeable working correlation structure to account for within-patient clustering of bilateral eye measurements. Continuous predictors (*Central* CT, Age, Rim Area) were centered at their respective medians for interpretability. The model adjusts for glaucoma severity, age, sex, rim area, and eye laterality as covariates. Coefficients represent population-averaged effects; p-values are based on robust standard errors. p_{adj} represents FDR-adjusted p-values using the Benjamini-Hochberg method across the models 10-18 for each parameter type. A p-value < 0.05 indicates statistical significance at the 5% level.

Table S.17. Generalized estimating equation model 15 results – association between *superior retinal nerve fiber layer thickness* and *superior corneal thickness* among glaucoma patients

Parameter	β	SE	95% CI	z	p	p_{adj}
Intercept	74.61	2.88	(68.96, 80.26)	25.88	**< 0.001**	**< 0.001**
Superior CT (centered at 607 μm)	0.01	0.04	(-0.06, 0.08)	0.24	0.814	0.915
Severe glaucoma (Yes vs. No)	-18.73	3.22	(-24.84, -12.62)	-6.01	**< 0.001**	**< 0.001**
Age (centered at 72 years)	-0.05	0.11	(-0.26, 0.16)	-0.50	0.614	0.915
Sex (Male vs. Female)	-0.46	2.85	(-6.05, 5.13)	-0.16	0.871	0.915
Rim Area (centered at 0.8 mm²)	6.54	3.04	(0.58, 12.49)	2.15	**0.032**	0.153
Eye (OS vs. OD)	3.02	1.97	(-0.84, 6.88)	1.53	0.125	0.915
Superior CT (centered at 607 μm) × Severe glaucoma (Yes vs. No)	0.05	0.05	(-0.04, 0.14)	1.02	0.306	0.915

Notes: SE = Standard Error; CI = Confidence Interval; RNFL = Retinal Nerve Fiber Layer; CT = Corneal Thickness; OD = Oculus Dexter (Right Eye); OS = Oculus Sinister (Left Eye). The outcome variable is *Superior RNFL thickness* (in μm). Model *15* was fitted to the glaucoma group using a GEE approach with an exchangeable working correlation structure to account for within-patient clustering of bilateral eye measurements. Continuous predictors (*Superior* CT, Age, Rim Area) were centered at their respective medians for interpretability. The model adjusts for glaucoma severity, age, sex, rim area, and eye laterality as covariates. Coefficients represent population-averaged effects; p-values are based on robust standard errors. p_{adj} represents FDR-adjusted p-values using the Benjamini-Hochberg method across the models 10-18 for each parameter type. A p-value < 0.05 indicates statistical significance at the 5% level.

Table S.18. Generalized estimating equation model 16 results – association between *temporal retinal nerve fiber layer thickness* and *temporal corneal thickness* among glaucoma patients

Parameter	β	SE	95% CI	z	p	p_{adj}
Intercept	60.14	2.85	(54.54, 65.73)	21.07	**< 0.001**	**< 0.001**
Temporal CT (centered at 562 μm)	-0.03	0.03	(-0.10, 0.03)	-1.00	0.317	0.915
Severe glaucoma (Yes vs. No)	-14.04	2.78	(-19.48, -8.60)	-5.06	**< 0.001**	**< 0.001**
Age (centered at 72 years)	-0.07	0.12	(-0.30, 0.16)	-0.60	0.548	0.915
Sex (Male vs. Female)	-2.17	2.83	(-7.72, 3.39)	-0.76	0.445	0.915
Rim Area (centered at 0.8 mm²)	0.77	2.09	(-3.34, 4.87)	0.37	0.714	0.915
Eye (OS vs. OD)	-0.39	1.79	(-3.89, 3.12)	-0.22	0.828	0.915
Temporal CT (centered at 562 μm) × Severe glaucoma (Yes vs. No)	0.03	0.05	(-0.06, 0.13)	0.66	0.510	0.915

Notes: SE = Standard Error; CI = Confidence Interval; RNFL = Retinal Nerve Fiber Layer; CT = Corneal Thickness; OD = Oculus Dexter (Right Eye); OS = Oculus Sinister (Left Eye). The outcome variable is *Temporal RNFL thickness* (in μm). Model *16* was fitted to the glaucoma group using a GEE approach with an exchangeable working correlation structure to account for within-patient clustering of bilateral eye measurements. Continuous predictors (*Temporal* CT, Age, Rim Area) were centered at their respective medians for interpretability. The model adjusts for glaucoma severity, age, sex, rim area, and eye laterality as covariates. Coefficients represent population-averaged effects; p-values are based on robust standard errors. p_{adj} represents FDR-adjusted p-values using the Benjamini-Hochberg method across the models 10-18 for each parameter type. A p-value < 0.05 indicates statistical significance at the 5% level.

Table S.19. Generalized estimating equation model 17 results – association between *inferior retinal nerve fiber layer thickness* and *inferior corneal thickness* among glaucoma patients

**Parameter**	**β**	**SE**	**95% CI**	**z**	**p**	**p_{adj}**
Intercept	76.51	3.45	(69.75, 83.26)	22.20	**< 0.001**	**< 0.001**
Inferior CT (centered at 582 µm)	0.06	0.05	(-0.04, 0.17)	1.16	0.247	0.915
Severe glaucoma (Yes vs. No)	-19.80	3.22	(-27.01, -12.60)	-5.39	**< 0.001**	**< 0.001**
Age (centered at 72 years)	-0.30	0.15	(-0.60, -0.01)	-2.03	**0.042**	0.153
Sex (Male vs. Female)	-1.47	2.81	(-6.98, 4.05)	-0.52	0.602	0.915
Rim Area (centered at 0.8 mm^2)	8.65	3.88	(1.05, 16.25)	2.23	**0.026**	0.153
Eye (OS vs. OD)	2.70	2.64	(-2.47, 7.87)	1.02	0.306	0.915
Inferior CT (centered at 582 µm) × Severe glaucoma (Yes vs. No)	-0.00	0.07	(-0.14, 0.13)	-0.07	0.945	0.945

Notes: SE = Standard Error; CI = Confidence Interval; RNFL = Retinal Nerve Fiber Layer; CT = Corneal Thickness; OD = Oculus Dexter (Right Eye); OS = Oculus Sinister (Left Eye). The outcome variable is *Inferior RNFL thickness* (in µm). Model *17* was fitted to the glaucoma group using a GEE approach with an exchangeable working correlation structure to account for within-patient clustering of bilateral eye measurements. Continuous predictors (*Inferior* CT, Age, Rim Area) were centered at their respective medians for interpretability. The model adjusts for glaucoma severity, age, sex, rim area, and eye laterality as covariates. Coefficients represent population-averaged effects; p-values are based on robust standard errors. p_{adj} represents FDR-adjusted p-values using the Benjamini-Hochberg method across the models 10-18 for each parameter type. A p-value < 0.05 indicates statistical significance at the 5% level.

Table S.20. Generalized estimating equation model 18 results – association between *nasal retinal nerve fiber layer thickness* and *nasal corneal thickness* among glaucoma patients

**Parameter**	**β**	**SE**	**95% CI**	**z**	**p**	**p_{adj}**
Intercept	64.63	2.20	(60.31, 68.95)	29.32	**< 0.001**	**< 0.001**
Nasal CT (centered at 588 µm)	-0.02	0.03	(-0.07, 0.03)	-0.76	0.447	0.915
Severe glaucoma (Yes vs. No)	-11.54	2.16	(-15.78, -7.30)	-5.33	**< 0.001**	**< 0.001**
Age (centered at 72 years)	-0.08	0.13	(-0.33, 0.17)	-0.62	0.535	0.915
Sex (Male vs. Female)	1.37	2.34	(-3.23, 5.96)	0.58	0.560	0.915
Rim Area (centered at 0.8 mm^2)	-2.31	2.72	(-7.64, 3.02)	-0.85	0.395	0.915
Eye (OS vs. OD)	-0.65	1.50	(-3.59, 2.30)	-0.43	0.668	0.915
Nasal CT (centered at 588 µm) × Severe glaucoma (Yes vs. No)	0.00	0.04	(-0.07, 0.07)	0.11	0.916	0.916

Notes: SE = Standard Error; CI = Confidence Interval; RNFL = Retinal Nerve Fiber Layer; CT = Corneal Thickness; OD = Oculus Dexter (Right Eye); OS = Oculus Sinister (Left Eye). The outcome variable is *Nasal RNFL thickness* (in µm). Model *18* was fitted to the glaucoma group using a GEE approach with an exchangeable working correlation structure to account for within-patient clustering of bilateral eye measurements. Continuous predictors (*Nasal* CT, Age, Rim Area) were centered at their respective medians for interpretability. The model adjusts for glaucoma severity, age, sex, rim area, and eye laterality as covariates. Coefficients represent population-averaged effects; p-values are based on robust standard errors. p_{adj} represents FDR-adjusted p-values using the Benjamini-Hochberg method across the models 10-18 for each parameter type. A p-value < 0.05 indicates statistical significance at the 5% level.

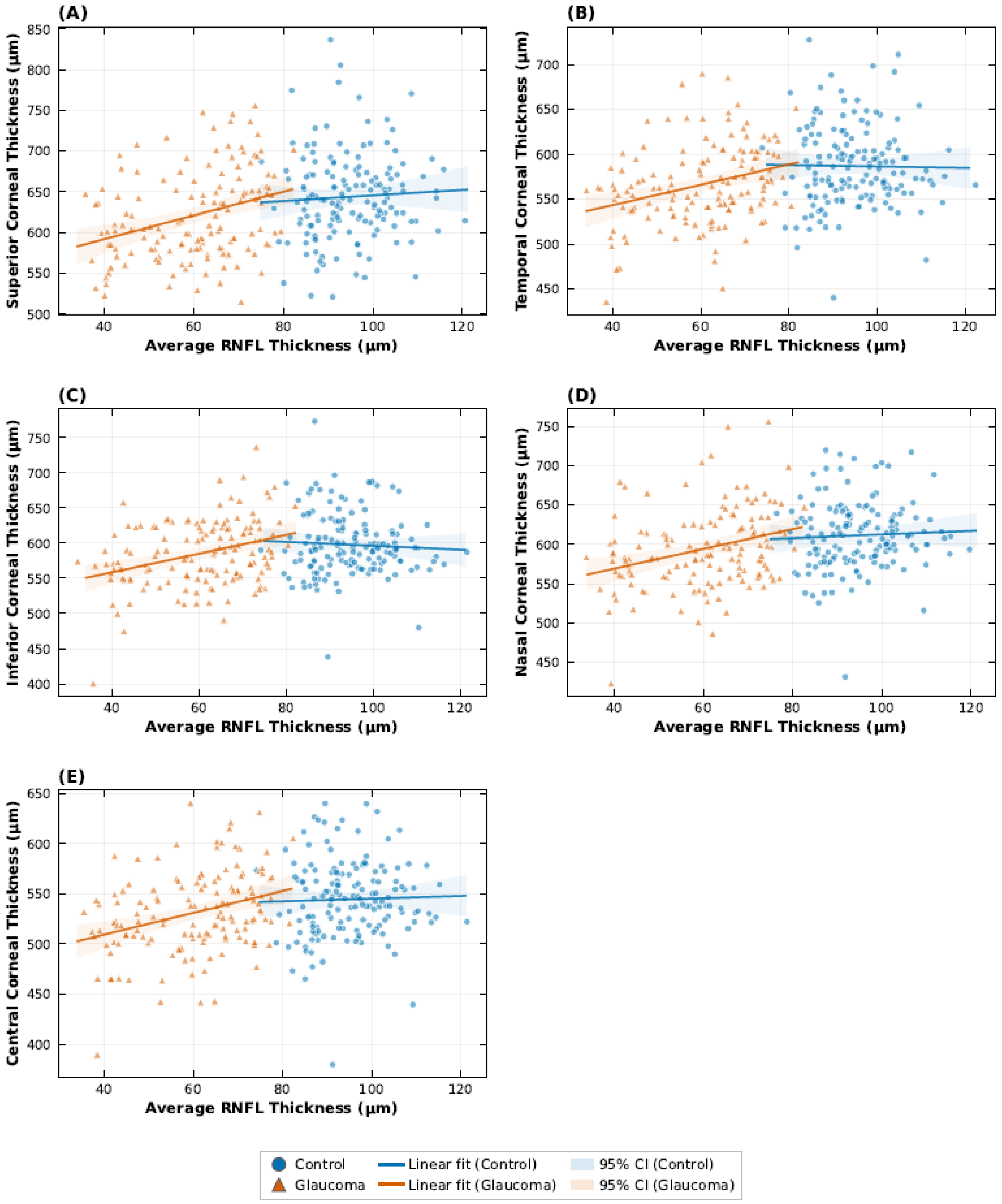

Figure S1. Scatterplots illustrating the relationship between average RNFL thickness and regional corneal thickness in glaucoma and control groups.

Panels depict average RNFL thickness plotted against (A) superior, (B) temporal, (C) inferior, (D) nasal, and (E) central corneal thickness. Group-specific ordinary least squares regression lines with corresponding 95% confidence intervals are shown.

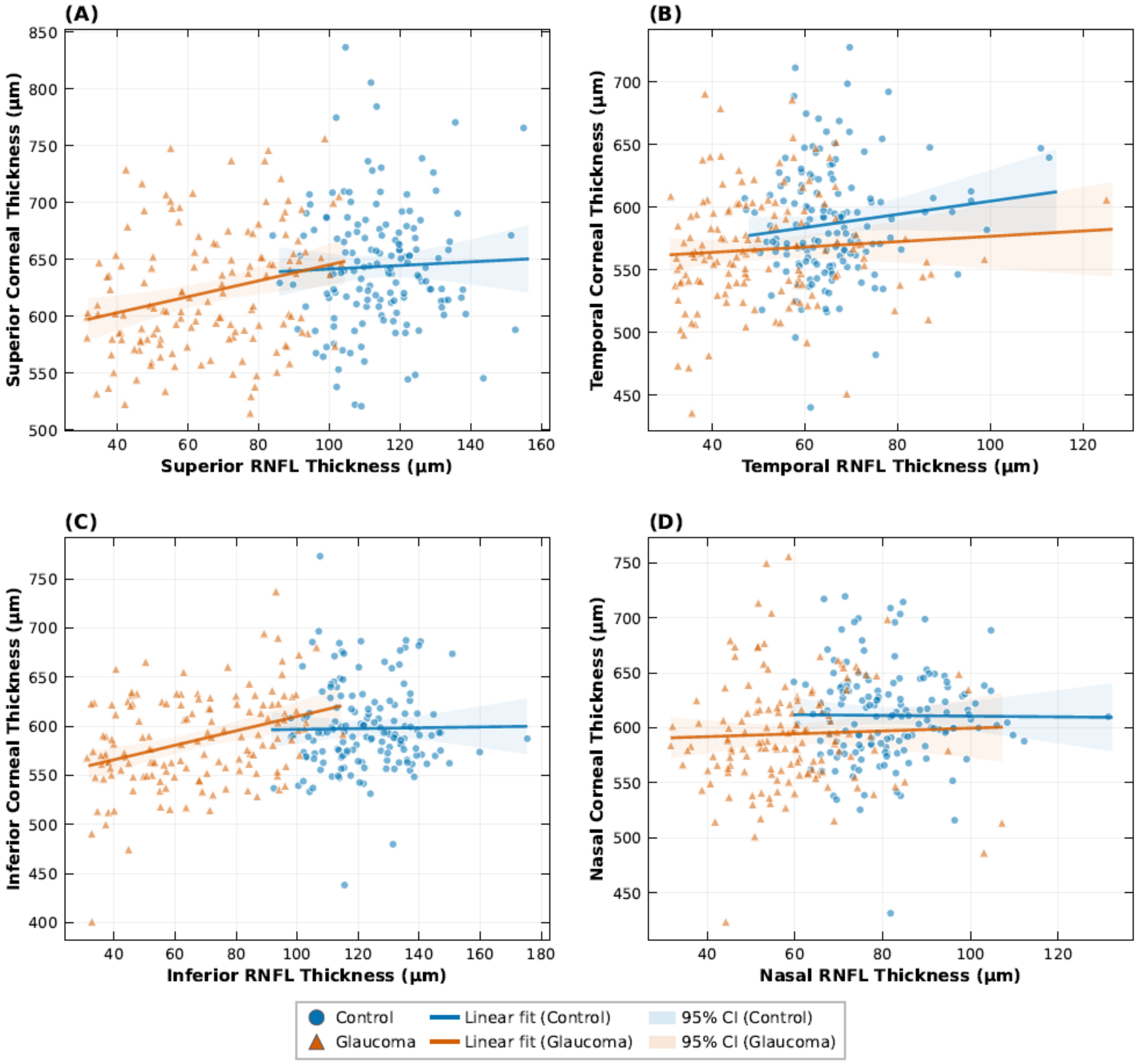

Figure S2. Scatterplots illustrating the relationship between quadrant-matched RNFL thickness and corresponding corneal thickness in glaucoma and control groups.

Panels depict (F) superior RNFL vs. superior corneal thickness, (G) temporal RNFL vs. temporal corneal thickness, (H) inferior RNFL vs. inferior corneal thickness, and (I) nasal RNFL vs. nasal corneal thickness. Group-specific ordinary least squares regression lines with corresponding 95% confidence intervals are shown.
